# Adrenal vein sampling in primary aldosteronism: a retrospective analysis of clinical experience in the Middle East

**DOI:** 10.1080/07853890.2025.2548973

**Published:** 2025-08-22

**Authors:** Fateen Ata, Arwa Ebrahim Y. A. Alsaud, Ali Ibrahim Rahil, Maab F. Elhaj, Ahmed Elmudathir Osman, Shafik Samir Mansour, Mohammad J. H. Elhissi, Ayman Elmagdoub, Ammara Bint I Bilal, Khaled Ahmed Baagar

**Affiliations:** a Department of Endocrinology, Hamad Medical Corporation, Doha, Qatar; b Department of Internal Medicine, Hamad Medical Corporation, Doha, Qatar; c Department of Radiology, Hamad Medical Corporation, Doha, Qatar

**Keywords:** Primary aldosteronism, hyperaldosteronism, adrenal vein sampling, Conn’s syndrome, secondary hypertension

## Abstract

**Context:**

Adrenal vein sampling (AVS) is the gold standard for lateralizing aldosterone secretion in primary aldosteronism (PA) management. However, due to challenges in expertise and infrastructure, it remains underutilized in the Middle East (ME).

**Objectives:**

This study aimed to document the performance and outcomes of AVS in the ME.

**Methods:**

This retrospective study included patients with PA who underwent AVS at a tertiary care hospital in Qatar (2015 – 2024). Clinical, biochemical, imaging and procedural data were collected to evaluate AVS success rates, imaging-AVS concordance and treatment outcomes. AVS success was defined as correct bilateral adrenal vein cannulation, determined by selectivity indices (unstimulated SI ≥ 2 or cosyntropin-stimulated SI ≥ 5).

**Results:**

The cohort (*n* = 31) had a mean age of 50.29 ± 8.19 years, with a male predominance (67.7%). AVS was successful in 71% (*n* = 22). There was a 59% concordance between AVS and adrenal imaging. Of those with successful AVS (*n* = 22), adrenalectomy was performed in 54.5% of patients, with 66.7% requiring fewer antihypertensives than pre-AVS. Pre-AVS hypokalemia resolved in 75% of patients undergoing AVS-guided adrenalectomies *vs.* 20% in the medically managed group.

**Conclusion:**

This study is the first to demonstrate AVS’s safety and efficacy in the ME, achieving a 71% success rate. With an AVS-imaging concordance of only 59%, the study highlights the clinical significance of AVS in guiding optimal management decisions. AVS-directed interventions lead to better treatment strategies with high post-treatment cure rates. The role of technology, such as intraprocedural dynaCT, is reinforced in improving procedural accuracy.

## Introduction

Primary aldosteronism (PA), a leading cause of secondary hypertension, is associated with high cardiovascular morbidity and mortality [1,2]. An accurate diagnosis followed by surgical removal of the culprit adrenal gland in patients with PA can potentially cure hypertension and its associated disease burden and improve quality of life [2,3]. Accurate differentiation between unilateral aldosterone-producing adenoma (APA) and bilateral adrenal hyperplasia (BAH) is imperative, as it guides treatment strategies, either surgical resection in the case of APA with clear lateralization on adrenal vein sampling (AVS) or medical therapy for BAH [4]. Patients who are eligible and opt for surgical resection of unilateral APA may require further testing to confirm laterality of aldosterone excess secretion in specific settings such as older age (>35 years) with unilateral adenoma on imaging, those with no adenoma on imaging or bilateral adenomas on imaging [5]. AVS has emerged as the gold standard in identifying the laterality of excess aldosterone secretion in these patient populations, leading to improved management and outcomes [5]. It is relatively safe if performed by experienced radiologists with a low record of local puncture site complications such as dissection, infarction, thrombosis and hematomas [4]. Rarely, adrenal hemorrhage or hematomas can develop after AVS [6]. High-volume centers have reported success rates exceeding 95% with adequately standardized procedures, establishing the potential for AVS to be highly reliable and clinically impactful [7].

Despite emerging as a cornerstone in optimal PA management, AVS still needs to be utilized in many parts of the world due to a lack of expertise and variable quality of healthcare infrastructure [8]. Limited data exist on AVS utilization in regions like the Middle East (ME), North Africa and South Asia (MENASA). This retrospective study evaluates AVS success rates and outcomes within an emerging healthcare landscape in the ME, aiming to establish benchmarks for procedural success and the clinical utility of AVS in the MENASA region.

## Research design and methods

### Study design and setting

**Figure 1.**
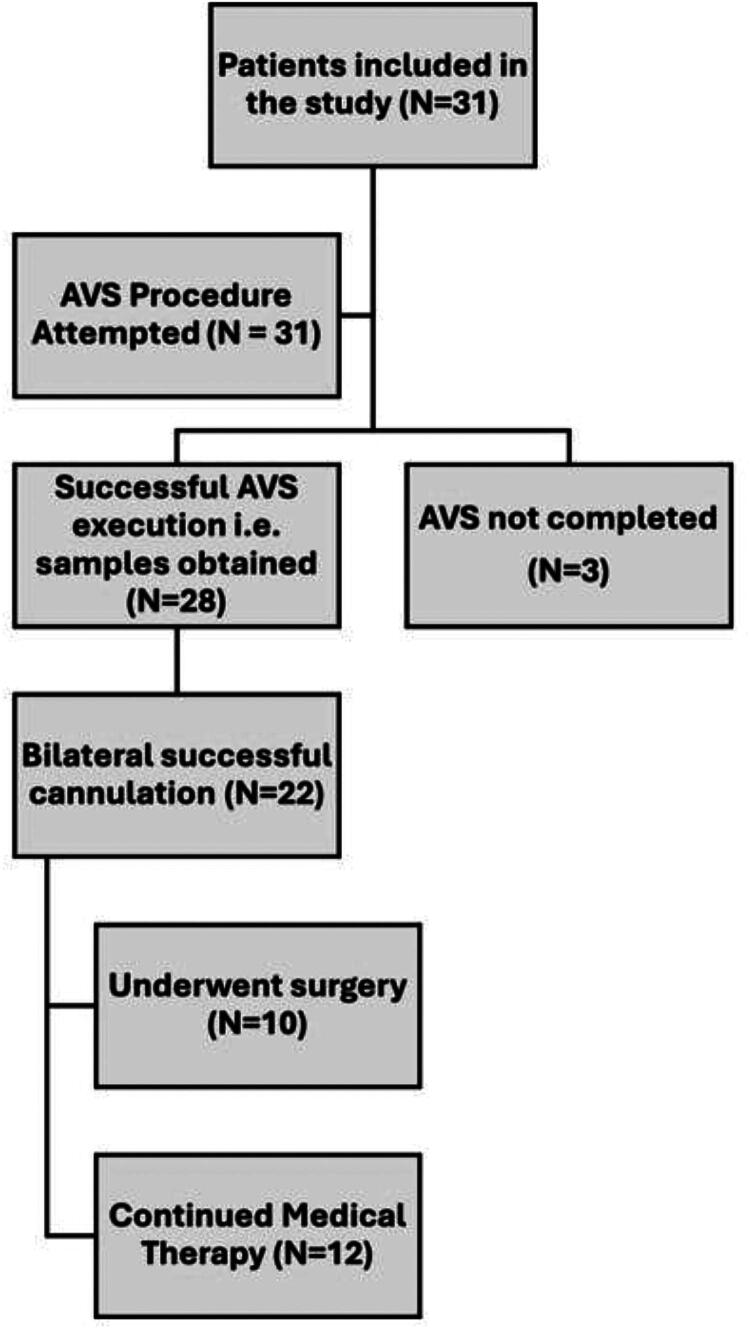
Patient inclusion and adrenal vein sampling (AVS) outcome stratification in the study cohort.

This retrospective study included 31 patients diagnosed with PA who underwent AVS at Hamad Medical Corporation (HMC) in Qatar between January 2015 and May 2024 (Figure 1).

### Definitions and inclusion/exclusion criteria

Inclusion criteria included patients aged >18 with biochemically confirmed PA who completed the AVS procedure. Patients with incomplete medical records, who underwent AVS without a confirmed PA diagnosis, or who could not complete the AVS procedure were excluded. PA diagnosis was considered accurate if the patients followed accurate case detection and confirmation. Patients with evidence of other secondary causes of hypertension, such as Cushing’s syndrome, pheochromocytoma and renovascular hypertension, were excluded. Pregnant patients were also excluded from the study, as they were not eligible for AVS according to the hospital’s AVS protocol.

Case detection was considered accurate if it was done for the following cases: patients with hypertension and hypokalemia, patients with persistent BP over 150/100 mm Hg, resistant hypertension despite treatment with at least three drugs including a diuretic, controlled BP on four drugs, hypertension with adrenal incidentaloma, hypertension with sleep apnea, hypertension with a family history of early hypertension or stroke before 40, hypertension with atrial fibrillation and hypertensive first-degree relatives of PA patient) and hypertension in young <40 years of age [5]. Screening was done with plasma aldosterone concentration (PAC), plasma renin activity (PRA) and aldosterone to renin ratio (ARR). Patients with PAC ≥ 277 pmol/L with PRA < 1 ng/mL/h were considered as having likely PA [9]. Patients with a PAC ≥ 555 pmol/L and PRA < 1 ng/mL/h with spontaneous hypokalemia were considered confirmed PA [9]. Those without this combination required a confirmatory test *via* saline infusion suppression test. If the PAC remained > 277 pmol/L after two liters of saline infusion over 4 h, patients were considered to have confirmed PA [10]. Successful catheterization of adrenal veins was defined by a selectivity index (SI) (adrenal vein to IVC cortisol ratios) of at least two without cosyntropin stimulation and at least 5 with cosyntropin stimulation [11 – 13]. The lateralization of aldosterone secretion was defined by an aldosterone/cortisol ratio on the culprit side at least four times higher than on the other side [9]. The contralateral suppression index (CSI) was calculated by dividing the aldosterone to cortisol ratio (ACR) of the nondominant adrenal by the inferior vena cava (IVC) ACR. A value of <1 was considered indicative of contralateral disease in cases with correct AVS cannulation bilaterally and <0.5 in cases with incorrect AVS cannulation [9].

### Data collection

Patient data were extracted from electronic medical records (Cerner), including demographic information (age, gender, ethnicity), clinical history (hypertension, hypokalemia, comorbidities), laboratory parameters (PAC, PRA, ARR, electrolytes), radiological findings (CT/MRI imaging of adrenal glands), AVS procedural details (aldosterone/cortisol ratios and use of cosyntropin) and post-procedure outcomes (lateralization results, AVS success rates, AVS complications, surgical outcomes and blood pressure [BP] control). The follow-up data were collected based on the most recent clinic visit within 24 months post-AVS.

#### AVS procedure

**Figure 2.**
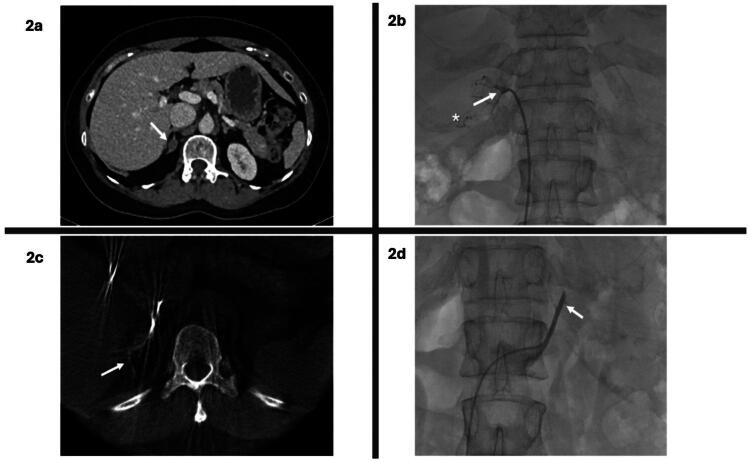
Multimodal imaging in adrenal vein sampling. (a) Computed tomography scan with IV contrast – venous phase showing right adrenal adenoma (arrow). (b) Right adrenal vein cannulation at the level of T12 confirmed with contrast enhancement of the right adrenal gland (arrow). Note emissary vein (asterisk). (c) DynaCT demonstrating enhancement of the right adrenal vein (arrow) during the AVS procedure. (d) the left adrenal vein common trunk (arrow) cannulation during AVS procedure.

The AVS protocol includes catheterizing both adrenal veins sequentially, confirming adrenal vein localization *via* cortisol levels, and calculating aldosterone-to-cortisol ratios. Pre-procedural imaging (CT or MRI scan of the adrenals) (Figure 2(a)) aids in identifying the site of the adenoma, drainage of the right adrenal vein, and potential anatomical variants of the left adrenal vein.

Patients with normal potassium are admitted on the same day in the daycare unit. Those below normal potassium are admitted one day before the AVS in the short admission unit for potassium correction. The procedure starts with the right common femoral vein puncture and 5 Fr vascular sheath insertion. To reduce the interval between the right and left adrenal vein cannulations, the right adrenal vein is cannulated first as it is usually more time-consuming (Figure 2(b)). Anatomical landmarks from pre-procedure cross-sectional imaging are used to localize the right adrenal vein, which usually drains directly into the right-posterior wall of the IVC at the level between the 11th thoracic vertebra and the 1st lumber vertebra. 4 Fr C2 Radifocus^™^ Optitorque^™^ (*Terumo*) catheter is the main catheter used for cannulation of the right adrenal vein. A side hole 2 – 3 mm from the tip of the catheter is done with a 22 G needle before the procedure to facilitate the blood extraction.

Once the right adrenal vein is engaged, DynaCT with an injection of 3 mL of diluted contrast is performed to confirm enhancement of the right adrenal gland (Figure 2(c)). Blood is usually extracted slowly using 5 ml syringes, and once extraction is complete, contrast injection is usually done to confirm the proper position of the catheter during the extraction. Enhancement of the right adrenal gland confirms correct proper adrenal vein engagement. The left adrenal vein usually shares a common trunk with the left inferior phrenic vein before draining into the superior aspect of the left renal vein. The common trunk is usually cannulated using a 4 Fr vertebral glide catheter, and blood is extracted from the common trunk to avoid supercannulation of the inferior phrenic vein (Figure 2(d)). Finally, samples are obtained from the IVC using the vertebral catheter or the vascular sheath.

During the study period, AVS procedures were performed by a team of two consultants (board-certified in both diagnostic and interventional radiology [IR]) and four fellows board-certified in diagnostic radiology and undergoing subspecialty training in the IR fellowship program.

### Study outcomes

The primary outcome included the success rate of the AVS procedure. Other outcomes comprised the concordance between AVS and imaging lateralization and AVS-guided management strategies. Other outcomes included the legibility of referral for AVS.

### Statistical analyses

Descriptive statistics summarize the cohort’s demographic and clinical data. Continuous variables are reported as means ± standard deviation or median (interquartile range) based on the distribution, while categorical variables are reported as numbers and percentages. All data are analyzed in STATA version 18 (StataCorp, College Station, TX).

### Ethical considerations

The study received ethical approval from the Medical Research Center of HMC (MRC-01-24-344). The IRB (Medical Research Centre, HMC, Qatar) waived the informed consent requirement because this is a retrospective study using existing records.

## Results

A total of 31 patients diagnosed with PA underwent AVS at our center during the study period (Figure 1). The baseline characteristics of the cohort are summarized in Table 1. The cohort had a mean age of 50.29 ± 8.19 years, with a male predominance (67.74%). Most patients were of Arab ethnicity (58.06%), followed by Asians (29.03%). The median duration of hypertension was 10 years (IQR: 7 – 18), with a median highest recorded systolic BP of 178.5 mmHg (IQR: 158 – 200). Notable comorbidities included chronic kidney disease (25.81%), diabetes mellitus (22.58%), dyslipidemia (22.58%) and coronary artery disease (12.9%).

**Table 1.** Baseline demographic and clinical characteristics of patients who underwent adrenal vein sampling after diagnosing primary aldosteronism (*n* = 31).

Variable	Results
Age (years)	50.29 ± 8.19
Gender	
Female	10 (32.26%)
Male	21 (67.74%)
Ethnicity	
Arabic	18 (58.06%)
Asian	9 (29.03%)
African	3 (9.7%)
Others	1 (3.2%)
Duration of HTN (years)	10 (7-18)
BMI kg/m^2^	30.67 ± 5.36
Highest systolic BP (mmHg)	178.5 (158 – 200)
Comorbidities	
CKD	8 (25.81%)
Diabetes	7 (22.58%)
Dyslipidemia	7 (22.58%)
CAD	4 (12.9%)
Stroke	2 (6.45%)
CLD	1 (3.23%)
Atrial fibrillation	1 (3.23%)

Data presented as mean ± SD, median (IQR) and numbers (%) as appropriate.

PA: primary aldosteronism; HTN: hypertension; BMI: body mass index; BP: blood pressure; CKD: chronic kidney disease; CAD: coronary artery disease; CLD: chronic liver disease

Results are reported as mean ± standard deviation, median (interquartile range) or number (percentage) as appropriate.

The biochemical, imaging and diagnostic characteristics of the cohort at the time of PA diagnosis are detailed in Table 2. The median PAC was 918 pmol/L (IQR: 742 – 1150), while the PRA was 0.14 ng/mL/h (IQR: 0.14 – 0.34), and ARR was 214.4 (IQR: 109.4 – 288.8). Hypokalemia was documented for 90.32% of patients. Confirmatory testing for PA diagnosis (*via* saline infusion) was required in 38.71% of patients. Imaging *via* CT or MRI revealed unilateral adrenal adenomas in 77.4% and bilateral adrenal adenomas in 12.9% and did not identify any adrenal adenoma in 9.7% of patients. The median adrenal adenoma size was 12 mm (IQR: 9 – 20).

**Table 2.** Biochemical, imaging and diagnostic characteristics of patients who underwent adrenal vein sampling at the time of diagnosis of primary aldosteronism (*n* = 31).

Variable	Results (*N* = 31)
PAC (pmol/L)	918 (742 – 1150)
PRA (ng/mL/h)	0.14 (0.14 – 0.34)
ARR (PAC converted to ng/dL to calculate ARR)	214.4 (109.4 – 288.8)
Hypokalemia	28 (90.32%)
Lowest potassium (mmol/L)	2.66 ± 0.57
PA confirmatory testing (saline infusion)	12 (38.71%)
Imaging	
CT Adrenals	16 (51.61%)
MRI Adrenals	15 (48.39%)
Size of an adrenal nodule in mm (largest one if bilateral/multiple)	12 (9 – 20)
Lateralization on imaging	
Bilateral adrenal lesions	4 (12.9%)
Left adrenal lesion	13 (41.9%)
Right adrenal lesion	11 (35.5%)
No adrenal lesion	3 (9.7%)

Data presented as mean ± SD, median (IQR) and numbers (%) as appropriate.

PAC: plasma aldosterone concentration; PRA: plasma renin activity; ARR: aldosterone-to-renin ratio; PA: primary aldosteronism; CT: computed tomography; MRI: magnetic resonance imaging Results are reported as mean ± standard deviation, median (interquartile range) or number (percentage) as appropriate.

**Figure 3.**
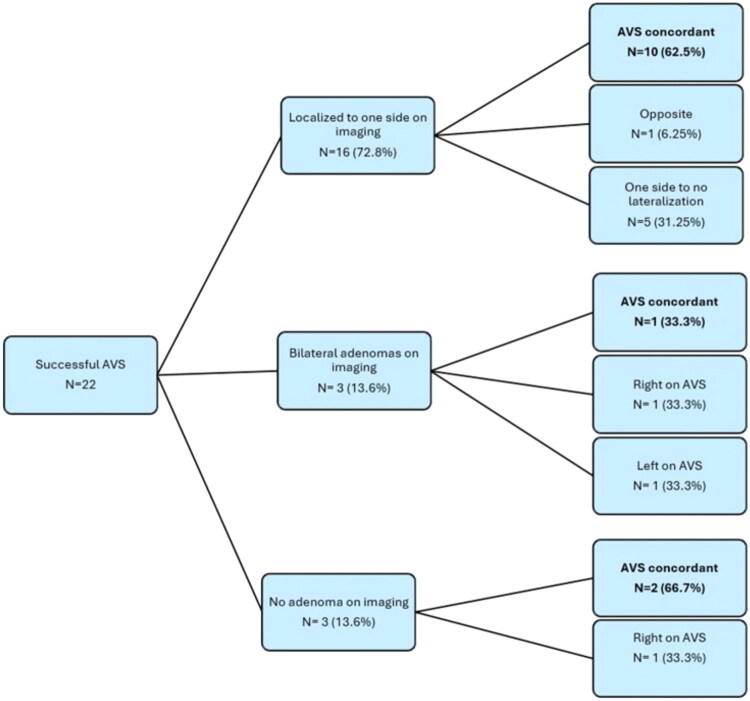
Concordance between imaging and adrenal vein sampling (AVS) for lateralization of aldosteronism in patients with primary aldosteronism.

Of the 31 patients, a prior AVS attempt was reported in 25.8% of patients (Table 3). Most patients underwent AVS during the study period under cosyntropin stimulation (87.1%). Of the 31 patients who underwent AVS, the procedure was successfully executed (with both adrenal veins cannulated and samples obtained) in 28 patients (90.3%), while it could not be completed in three patients (9.7%) due to technical challenges (inability to cannulate the veins and acquire aldosterone/cortisol samples). Correct bilateral cannulation (determined by SI) was documented in 22 patients (71%). Based on the selectivity indices, incorrect cannulation occurred on the left side in two cases and on the right in three, whereas both sides were incorrectly cannulated in one patient. Of these, three patients had a concordance with the imaging based on the CSI being suppressed more than IVC (0.26, 0.15 and 0.06). Among the 22 patients with successful AVS, concordance between imaging and AVS was observed in 59% of cases. Among the patients with unilateral adenomas on imaging, 62.5% showed concordant lateralization, while 33.3% of those with bilateral adenomas and 66.7% of those without adrenal lesions had non-lateralizing AVS. These distributions are further illustrated in Figure 3.

**Table 3.** Adrenal vein sampling procedure details in the total cohort (*n* = 31).

Variable	Results
AVS tried before	8 (25.8%)
Cosyntropin stimulation	27 (87.1%)
Procedure executed (both sides cannulated physically and samples taken)	28 (90.3%)
Procedure failed	3 (9.7%)
SI results (*N* = 28)	
Correct cannulation bilaterally	22 (78.57%)
Left incorrect cannulation	2 (7.14%)
Right incorrect cannulation	3 (10.71%)
Bilaterally incorrect cannulation	1 (3.57%)

AVS: adrenal vein sampling; SI: selectivity index

Table 4 details the biochemical measurements, selectivity, and lateralization outcomes from successful AVS cases (*n* = 22). The median SI was 10.87 (IQR: 8.14 – 19.7) on the left and 20.4 (IQR: 16.55 – 32.2) on the right. The median lateralization index was 6.81, whereas the median CSI was 0.155 (IQR: 0.06 – 0.7).

**Table 4.** Biochemical measurements, selectivity, and lateralization outcomes from successful adrenal vein sampling cases with correct cannulation (*n* = 22).

Variable	Results
Cortisol nmol/L	
Left AV	8549 (5363 – 14,012)
Right AV	16,014 (12,866 – 17,500)
IVC	778 (636 – 844)
Selectivity index	
Left AV	10.87 (8.14 – 19.7)
Right AV	20.4 (16.55 – 32.2)
Aldosterone (absolute values) pmol/L	
Left AV	22,650 (3140 – 27,740)
Right AV	15,900 (2630 – 27,740)
IVC	1400 (943 – 2050)
Lateralization based on AVS	
Median lateralization index	6.81 (2.33 – 16.5)
No lateralization	8 (36.36%)
Left	8 (36.36%)
Right	6 (27.27%)
Contralateral suppression Index	
Median CSI	0.155 (0.06 – 0.7)
<1	19 (86.36%)
>1	3 (13.64%)

AVS: adrenal vein sampling; AV: adrenal vein; IVC: inferior vena cava; CSI; contralateral suppression index

Results are reported as median (interquartile range) or number (percentage) as appropriate. SI ≥ 2 for non-stimulated and ≥5 for cosyntropin-stimulated samples defines successful catheterization.

The median length of stay for AVS was 1 day (1.2). AVS-guided management planning led to surgery in 12 out of 22 patients, with the rest continued on medical management. Of the 10 continued on medical management, two awaited surgery at the time of data extraction. Table 5 compares the outcomes of patients based on the type of post-AVS management (medical *vs.* surgical). Patients were grouped based on the actual management received post-AVS, considering that clinical decisions may not always align with AVS findings due to individual considerations. Systolic BP at follow-up in the medically managed group was 139.5 mmHg (120 – 152) compared to 131.5 mmHg (123 – 144) in the surgically managed group. Similarly, the median diastolic BP was 85 mmHg (80 – 89) in the medically managed group and 84.5 mmHg (73 – 88) in the surgically managed group. The median number of anti-hypertensives in the medically managed group was 4 (3 – 4) compared to 1 (1 – 2) in the surgical group. Hypokalemia resolved in 20% of patients in the medically managed group and 75% in the surgically managed group.

**Table 5.** Clinical outcomes and treatment decisions following successful adrenal vein sampling (AVS) in 22 patients.

Variable	Medical management 10 (45.5%)	Surgical management 12 (54.5%)
Systolic BP at last FU (mmHg)	139.5 (120 – 152)	131.5 (123 – 144)
Diastolic BP at last FU (mmHg)	85 (80 – 89)	84.5 (73 – 88)
Number of anti-HTN drugs before AVS	3.5 (3 – 4)	2.5 (1.5 – 3.5)
Number of anti-HTN drugs after AVS-based management	4 (3 – 4)	1 (1 – 2)
Anti-HTN medication detail at last FU (*N* = 22)		
Eliminated	0	2 (16.7%)
Reduced	0	6 (50%)
Same number drugs	8 (80%)	2 (16.7%)
Increased	2 (20%)	0
Lost to FU	0	2 (16.7%)
Hypokalemia at last FU		
Resolved	2 (20%)	9 (75%)
Persistent	6 (60%)	0
Was never present	2 (20%)	1 (8.3%)
Patients lost to FU	0	2 (16.7%)

Results are reported as median (interquartile range) or number (percentage) as appropriate.

AVS: adrenal vein sampling; PA: primary aldosteronism; LOS: length of stay; BP: blood pressure; FU: follow-up; Anti HTN: antihypertensive

## Discussion

This retrospective study highlights the clinical implications of AVS as a lateralization procedure for patients with PA in the ME, where data on AVS success rates and utilization have not been reported before to the best of our knowledge. We demonstrated an AVS success rate of 71%. Among patients with successful AVS, the results demonstrated concordance with imaging in specific subsets: 62.5% of patients with unilateral adenomas on imaging showed a lateralization on AVS. In comparison, 33.3% of those with bilateral adenomas on imaging demonstrated no laterality on AVS. Additionally, in patients without an adenoma on imaging, 66.7% showed no lateralization on AVS, highlighting the role of AVS in effectively ruling out a tiny unilateral secreting adrenal lesion that could be otherwise missed on imaging. AVS-based management led to surgery in 63.6% of patients. A reduction or elimination of anti-hypertensive drugs was observed in 37% of patients, whereas hypokalaemia resolution was reported in 88%.

Adrenal imaging is a valuable and accurate tool to localize the source of most adrenal hormonal excess disorders, like ACTH-independent hypercortisolism and pheochromocytoma. However, its accuracy is limited when localizing the source of hyperaldosteronism. This has become increasingly evident as more data on AVS-imaging concordance continues to emerge. For instance, Cartwright et al. studied 21 patients with PA who underwent AVS followed by adrenalectomy, finding that 40% of cases displayed discordance between imaging and AVS results [14]. Nwariaku et al. reported only 54% concordance between computed tomography (CT) and AVS results in 41 patients with PA [15]. From the successful AVS results of 194 patients, Young WF et al. concluded that based on CT findings alone, 42 patients (21.7%) would have been incorrectly excluded as candidates for adrenalectomy, and 48 (24.7%) might have had unnecessary or inappropriate adrenalectomies [16]. These findings underscore the clinical relevance of AVS in achieving optimal PA management. Most expert guidelines (including Endocrine Society clinical practice guidelines) advocate for AVS in localizing the source of hyperaldosteronism in most surgical candidates [12]. A meta-analysis of 950 patients with PA who underwent AVS reiterated the fact that a notable proportion (37.8%) of patients exhibit discordance between imaging and AVS laterality [17]. Our study further highlights the variability between adrenal imaging and AVS findings in a previously unexplored patient population **(**Figure 3). We found an overall AVS and imaging concordance in only 59% of patients. Given the significant discordance, especially in cases with nonlocalized adenoma on imaging, AVS should remain the standard method for determining lateralization in surgical candidates with PA to improve clinical outcomes.

AVS success rates are highly dependent on patient factors (such as age, anatomy of adrenal veins) and external factors (such as center resources, use of standard protocols, physician expertise and procedural preferences) [18,19]. Reported AVS success rates range from 41% to 99%, with an average success rate of 74% [18,19]. We have also reported a comparable AVS success rate (71%). The majority of patients in our cohort had AVS done under cosyntropin infusion (87%), which is known to increase the success rates of AVS by at least four times [11,19]. Intraprocedural imaging such as cone-beam and dynaCT scan has been shown to increase procedural success rates by confirming the cannulation of adrenal veins [11,20]. While procedural success in this study appeared to improve following the adoption of intraprocedural dynaCT, this observation is based on clinical impression (AVS done before dynaCT introduction in the center had more failure rates) rather than statistical analysis, as the small sample size and absence of a pre-defined comparator group limited a formal evaluation. Intraprocedural CT provides real-time visualization of adrenal anatomy, enhancing successful catheter placement and significantly reducing inaccurate sampling [20,21]. Precise imaging of the adrenal veins during the AVS aids in confirming cannulation, distinguishing the adrenal vein from nearby structures like accessory hepatic veins, and identifying anatomical variants in adrenal veins. This is most useful in right adrenal vein cannulation, which is inherently difficult due to its short length, variable entry angles and proximity to the IVC [11,21].

In our cohort, AVS was done using a rapid sequential cannulation approach rather than simultaneous cannulation. Some studies have shown improved success rates with the simultaneous cannulation approach, but the evidence is still weak to make definitive conclusions [22]. Studies actively comparing both techniques have reported similar efficacy [23]. The sequential approach requires cosyntropin infusion and immediate cannulation of the subsequent veins (ideally within 5 min) for comparable results [23]. Two approaches are commonly encountered in how interventional radiologists (IR) perform AVS. The first involves a single highly experienced IR performing all AVS procedures, which results in success rates approaching 98% [24]. While this approach ensures consistency in procedural success, it limits training and capacity-building, which are integral to the globalization of AVS. The second approach, often adopted in teaching institutions, focuses on the dissemination of skillsets by involving multiple IR physicians in performing AVS under the supervision of an experienced radiologist [25]. While this model may initially yield relatively lower success rates secondary to IR physicians’ variable expertise, it enhances the broadening of expertise across the department [26]. Our center, being a large tertiary care teaching hospital, utilized the second approach, with success rates similar to or more than studies reporting multiple IR physicians’ experiences [25,26]Our center, which has a very diverse healthcare staff, advocates for this approach, aligning with the long-term goal of expanding AVS procedural expertise globally to address the unmet need in the MENASA region, where AVS expertise remains limited. At the same time, this approach enriches trainees’ clinical experience in this academic teaching institution.

Compared to essential hypertension, PA is associated with significantly higher risks of adverse health outcomes. These include an increased odds of developing atrial fibrillation (odds ratio [OR] 3.52), stroke (OR 2.58), left ventricular hypertrophy (OR 2.29), heart failure (OR 2.05), coronary artery disease (OR 1.77), metabolic syndrome (OR 1.53) and diabetes (OR 1.33) among other risks [5]. These comorbid conditions are the primary drivers of poor health in most of the world’s population and the significant influencers of healthcare resource utilization, making optimal management of PA imperative in providing high-quality care. AVS has played an integral role in optimizing clinical decisions for PA management, improving the outcomes multifold [27]. Studies focused on AVS-guided clinical outcomes in patients with PA have shown around 35% complete cure and 47.5% partial clinical recovery from PA [27]. In our cohort of patients with bilaterally successful AVS (*n* = 22) who underwent surgery, clinical recovery (in terms of complete resolution of hypertension indicated by elimination of pre-AVS antihypertensives) was observed in around 17%. In contrast, partial recovery was seen in 50% (indicated by a reduction in antihypertensives). However, hypokalemia resolution was seen in a significant majority of those who underwent adrenalectomy based on AVS (75%). Some variability in our results is expected due to multiple factors influencing clinical recovery after surgical treatment for PA. These include the surgeon’s expertise, concomitant essential hypertension (unfolding or emerging post-PA management), and demographic, genetic and environmental differences among patient cohorts, among other factors [28]. Additionally, certain comorbid conditions, like diabetes mellitus, are differently prevalent in various parts of the world and can affect the outcomes in patients with PA undergoing surgical management [29]. These multifactorial influences highlight the challenges in predicting outcomes in PA and hence stress the need to optimize clinical decision-making to choose the patients that would benefit the most from surgery, with AVS playing one of the most critical roles in pre-operative planning.

The primary strength of this study lies in its pioneering presentation of real-world data on the safety and effectiveness of AVS in the ME. Success rates are comparable to international data, with no notable complications. Guidelines-based case detection and confirmation approaches allowed accurate representation of patients with PA. The multiethnic population of Qatar allowed representation from Arabic and Asian patients with PA. However, the study contains some notable limitations to consider when interpreting our findings. These include the retrospective nature of data collection and associated biases, such as sampling bias. Some data was unavailable (such as post-surgery biochemical tests including aldosterone and renin) and the small sample size did not allow us to use imputation for the missing data. The sample size was also insufficient for advanced statistical analyses and limited our results’ interpretation. For instance, after stratifying the participants into surgical and medical subgroups, the sample size limited the feasibility of meaningful statistical comparisons between groups. Loss to follow-up, although below 10%, might have influenced results. Our study was retrospective in nature, and hence, it had variable follow-up visits, which may have influenced the accuracy and comparability of post-treatment BP outcomes. While pregnant patients were excluded from the study, individuals with recent cardiovascular events, surgery, or active malignancy were not excluded. These conditions may transiently influence PAC and could have introduced variability in baseline PAC measurements. Nevertheless, the study provided significant information on the utilization of AVS in the region and will serve as a benchmark for further research on AVS and PA in the region.

## Conclusion

This study is the first to document AVS’s safe and effective utilization in patients with PA within a Middle Eastern cohort. Our AVS success rate of 71% aligns with international data. With an AVS-imaging concordance of only 59%, the study also reinforces the clinical significance of AVS in guiding optimal management decisions in the era of precision medicine. AVS-directed interventions lead to better treatment strategies with high post-treatment cure rates. This study also highlights the role of technology, such as intraprocedural dynaCT, in improving procedural accuracy and, ultimately, patient care. More research on AVS from previously unexplored cohorts is imperative to address the challenges in its globalization.

## Data Availability

Some or all datasets generated during and/or analyzed during the current study are not publicly available but are available from the corresponding author on reasonable request.
